# Educational design research: Portraying, conducting, and enhancing productive scholarship

**DOI:** 10.1111/medu.14280

**Published:** 2020-08-05

**Authors:** Susan McKenney, Thomas C. Reeves

**Affiliations:** ^1^ Department of Teacher Development (ELAN) Faculty of Behavioural, Management and Social Sciences University of Twente Enschede the Netherlands; ^2^ Department of Career and Information Studies College of Education University of Georgia Athens Georgia USA

## Abstract

**Context:**

Solutionism is the all‐too‐common human propensity to jump to a solution before adequately understanding the nature of a problem. Solutionism has long been prevalent in efforts to improve education at all levels, including medical education.

**Thesis:**

Educational design research (EDR) is a genre of research that features the gaining of in‐depth understanding of a problem before any prototype solution is designed and tested. It is different from other forms of scientific inquiry because it is committed to the simultaneous development of both theoretical insights and practical solutions, together with stakeholders. This approach is powerful for theory building because it privileges ecologically valid studies that embrace the complexity of investigating learning in authentic (as opposed to laboratory) settings. When conducted well, both the research process and its outcomes generate valuable contributions to practice.

**Preview:**

This article constitutes an expository essay on EDR, comprised of three movements. First, the approach is defined, its origins are presented, and its characteristics are described. Second, a generic model for conducting EDR is offered, and illustrated with examples from the field of medical education. Third, pathways towards advancing this form of inquiry are discussed, including ways to address inherent challenges and limitations, as well as recommendations for the medical education community. Although EDR is no panacea, this article illustrates how it can serve medical education research in a wide variety of geographic and disciplinary contexts.

**Key message**

Educational design research features collaboration between stakeholders (eg, researchers, instructors, clinicians, and medical students) to simultaneously develop both new theoretical insights and practical solutions to serious teaching and learning challenges.

## 1 INTRODUCTION

Humans have a common tendency to jump to solutions prior to fully understanding the nature of the problem they are trying to solve, thus demonstrating a practice described as ‘solutionism’ by Morozov.^1^ Education is not the only field to fall prey to such behaviour, but it has seen its fair share of examples over the course of many decades. For instance, in 1922 Edison promised that films would replace textbooks.^2^ Similar predictions continue to be made today as augmented reality and other technologies are put forward as the future of medical education.^3^ Despite the fact that critics have revealed that the majority of studies on technological solutions in education yield ‘no significant difference,'^4^ the tendency persists. Perhaps this represents the triumph of optimism over experience, or perhaps it is simply naïveté. We posit another explanation, which is that for many years problem solving was not considered serious science. In this contribution to *Medical Education*’s special issue on ‘solutionism,' we examine a powerful approach to education research that features productive synergies between problem solving and serious science.

A distinction has often been made between basic research to discover new knowledge and applied research to solve practical problems, but this simplistic dichotomy does not adequately represent either how research is actually conducted or the multiple goals pursued by most scholars.^5^ Education researchers, including those working in the field of medical education, often have a range of different goals in that their purposes may be descriptive, predictive, interpretivist, or refer to development or action.^4^ For researchers interested in contributing to theory alongside development goals, educational design research (EDR) may be a compelling option.^6^ We observe that the achieving of complex development goals is rarely feasible through simple, linear or predictable pathways, and this is also the case in medical education.

## 2 PORTRAYING EDR

‘Educational design research can be defined as a genre of research in which the iterative development of solutions to practical and complex educational problems also provides the context for empirical investigation, which yields theoretical understanding that can inform the work of others.’^7^ Identifying problems amenable to EDR involves finding real‐world challenges that are worthy of investigation and capable of being solved through the EDR process. Specific problems may be identified by practitioners, by researchers, or through the study of literature.^7^ For example, a serious problem in medical education refers to helping future physicians develop consistent habits to prevent sepsis.^8^ Over a million cases of sepsis occur in United States hospitals every year and 15%‐30% of them result in death.^9^ Although antisepsis protocols are well known, research is needed to understand why they are insufficiently adhered to, and to develop additional solutions that can eradicate this problem.^10^

When even an obvious serious problem is raised, verification in both literature and practice is necessary to ascertain if it is, indeed, legitimate, researchable and research‐worthy. From the theoretical perspective, the problem is worth studying if doing so would address a clear gap in the existing literature (legitimate), if existing methods will allow it to be studied well enough to warrant the effort (researchable), and if the work will contribute to theory development or scientific understanding related to a widely held, as opposed to idiosyncratic, concern (research‐worthy).^7^ From the practical perspective, the problem is worth solving if the real problem, as opposed to a symptom, is identified (legitimate), if it can be identified in accessible contexts (researchable), and if it is severe enough to encourage stakeholders to invest in solving it (research‐worthy).^7^ Here is an example situated in a gross anatomy class:

- Problem: corpse donors are limited and existing simulations for teaching human anatomy lack sufficient fidelity (the practical side of the problem). The current simulation literature provides insufficient guidance on how to develop high‐quality simulations for mammalian anatomy (the scientific side of the problem).
- Practical aim: to develop a high‐quality human dissection simulation that allows students to meet course goals without conducting actual dissection.
- Scientific aim: to understand and describe the characteristics of high‐quality simulations for mammals in general and humans in particular.

Research that does not explicitly seek to contribute to both theory and practice by addressing real‐world challenges can certainly be of great value, but it does not constitute EDR.

Undertaken in three movements, the purpose of this paper is to introduce EDR to the medical education community. The remainder of this section further portrays (the origins of) the approach, as well as similar approaches found in medical education research. The second section of this article describes how EDR is conducted and gives examples from the field. The third section considers productive pathways forward, in light of inherent challenges and limitations.

### 2.1 Theoretical and practical synergies: a brief, non‐comprehensive review

The notion of a linking science connecting theoretical and practical work has been advocated by psychologists for over a century.^11^, ^12^ In the 1930s and 1940s, major advancements in this direction were made by Lewin and colleagues, through action research, in which hypothesis generation and testing through the discussion of problems followed by group decisions were central.^13^ In the 1960s and 1970s, (participatory) action research flourished in the social sciences, practitioner inquiry emerged, and calls for educational research to directly address the problems and needs of education increased.^14^ Amongst other things, this set the stage for the rise of ‘action science’ in the 1980s^15^ and the notion of ‘use‐inspired basic research,' which gained widespread momentum in the 1990s^5^. Around that time, researchers in the fields of instruction design and curriculum development began to stress the need for more reliable, prescriptive understanding to guide the robust design of educational products, programmes, processes and policies.^16^, ^17^, ^18^ At the same time, researchers in the field of education psychology published landmark papers arguing for how theory informs the design of learning and vice versa, calling for research to be situated in the contexts in which that learning actually takes place, and citing the shortcomings of laboratory settings for understanding learning phenomena.^19^, ^20^

Across disciplinary lines, such views gained momentum upon the publication of *Pasteur’s Quadrant: Basic Science and Technological Innovation*.^5^ In this seminal work, Stokes questions the popular assumption that basic research inevitably leads to the development of new technologies, and argues that technological advances often permit the conduct of new types of basic research, thus reversing the direction of the basic to applied model. Moreover, he argues for more research like that of the French chemist and microbiologist Louis Pasteur, who sought fundamental knowledge within the context of solving real‐world problems such as the spoilage of milk and treatment for rabies. In this tradition, EDR is concerned with the solving of existing problems in practice and with the structuring of the inquiry process so that it yields scientific understanding that is ecologically valid and informs the work of others.

### 2.2 A family of approaches

We use the term 'EDR' to describe a family of approaches that strive towards the dual goals of developing theoretical understanding and also designing and implementing interventions in practice.^21^ This family of approaches may, but does not always, include design‐based research, design‐based implementation research, development research, design experiments, formative research, participatory design research, realist evaluation, the Medical Research Council (MRC) framework for evaluating complex interventions, action research and improvement science. The various names are not synonymous, and some authors have extensively described how specific members of this family differ from others.^22^ Although a comprehensive overview is beyond the scope of this contribution, we do attempt to situate EDR in relation to other approaches that are frequently used in (medical) education research by way of Table 1. Though citing the literature upon which it is based, Table 1 summarises the goals and characteristics of each approach in light of that which sets EDR apart from other forms of inquiry: the pursuit of theoretical understanding through the (iterative) development of solutions to problems in practice. Cells with grey shading indicate approaches that inherently yield theoretical and practical outcomes through intervention development, whereas white cells indicate that both theoretical and practical outcomes may be sought through the given approach, but this is not necessarily the case.

**TABLE 1:** Family of approaches seeking practical and scientific synergies, including those that inherently yield theoretical and practical outcomes through intervention development (grey cells), and those that may yield theoretical and practical outcomes, depending on how they are used (white cells)

Approach	Goals	Key characteristics
Design‐based research (DBR)^6^, ^44^, ^49^	To enhance understanding about the nature of learning and what facilitates it	Takes place in continuous cycles of design, evaluation and redesign Takes place in authentic real‐life learning settings in which learning occurs normally Is aimed at both testing and refining theories, and advancing practice Is characterised by mixed‐methods studies Involves designers, researchers and practitioners with different expertise who interact frequently to guide the design, conduct and reporting of DBR
Design‐based implementation research (DBIR)^22^, ^50^	To address differences (both positive and negative) between innovative interventions as designed and as they are actually implemented in practice	Focuses on persistent problems of practice from multiple stakeholders’ perspectives Commits to iterative, collaborative design Develops theory and knowledge related to both classroom learning and implementation through systematic inquiry Develops capacity for sustaining change in systems
Development research^17^, ^51^	To enhance interventions through iterative, scientific testing and refinement	Optimises curricular interventions (eg, curriculum frameworks, curriculum materials, policies and programmes) Defines and refines curriculum design principles Fosters professional development of practitioners, researchers and other stakeholders
Design experiments^19^, ^20^, ^52^	To create and test particular models of learning within real‐world contexts	Explores how the pragmatic and theoretical aspects of innovative models of learning function in real‐world contexts Intervenes to support new models of learning Develops and refines humble (rather than bold) theories that have clear implications for practice
Formative research^16^, ^53^, ^54^	To understand how instructional innovations can be optimised to achieve specific instruction goals	Articulates the pedagogic goal of the experiment Describes underlying pedagogic theory Describes how innovations can potentially achieve the pedagogic goals Investigates what factors enhance or inhibit effectiveness Hypothesises how innovations and implementations can be enhanced to optimise effectiveness Refines underlying theories accordingly
Realist evaluation^55^	To establish what works, for whom, in what circumstances, in what respects, to what extent, and why	Is informed by theory Seeks to test and refine the theory underlying an intervention Seeks to determine the outcomes of the intervention Helps make decisions about the adoption or dissemination of interventions
Participatory design research (PDR)^50^, ^56^	To critique and deconstruct power inequities in society with the design of practical solutions to serious problems underlying such inequities	Advances fundamental insights about human learning and development Exposes explicit or implicit normative hierarchically powered decision‐making structures and related assumptions of objectivity Critically attends to a range of theoretical lenses (eg, colonial, racialised, gendered, queered) during design and partnering
Medical Research Council (MRC) framework^57^	To employ experimental methods as well as dealing with the complexity inherent in public health and education innovations	Develops evidence base, theory, models for processes and outcomes Pilots with attention to feasibility, recruitment and retention, sampling Evaluates to assess effectiveness, understand change processes, assess cost‐effectiveness Implements with attention to dissemination, monitoring and follow‐up
Action research^58^, ^59^	To address problematic situations in organisations or communities	Is conducted by insiders of organisations or communities Starts with goal setting Explores relevant theory and practice Identifies research questions Involves data collection, analysis and reporting Results in the taking of informed action
Improvement science^60^, ^61^	To explore how to undertake quality improvement well	Makes the work problem‐centred Focuses on variation in performance as central to the problem Sees the system that produces the current outcomes Values measurement as crucial for improvements at scale Is anchored in cycles of disciplined inquiry (plan, do, study, act) Accelerates improvements through networked communities

### 2.3 Examples of EDR in medical education

Like other research, EDR extends existing theoretical knowledge through data collection and analysis.^23^, ^24^, ^25^ However, unlike many other kinds of research, the EDR process is embedded in the (often cyclic) development of a solution to the problem being tackled.^23^, ^24^, ^25^ Here are three examples:

- Hege et al^26^ describe the research embedded in the development of a clinical reasoning tool featuring virtual patients. After a framework for software design had been developed based on psychological theories, patient‐centredness, teaching and assessment, learner‐centredness and context, a software tool was developed to be used with virtual patient systems. It specifically supports clinical reasoning skills acquisition and assesses all steps of this complex process. The authors^26^ describe the main components of the software, results of usability tests and a pilot study, and indicate directions for further development.
- Duitsman et al^27^ describe their cyclic approach to improving the functioning of a clinical competency committee. In this project, theoretical principles were distilled from the literature to (re‐)shape clinical competency meetings focused on resident performance in a university children’s hospital. The meetings were evaluated and deemed useful for obtaining a broad indication of resident performance. The design principles and recommendations given are useful for other (research on) residency programmes, and can be adjusted to different contexts.
- Baarends et al^28^ conducted EDR to foster evidence‐based decision‐making capacity amongst undergraduate occupational therapy students. Their report describes a mixed‐methods study undertaken to generate and pilot test the pedagogic principles underlying a teaching and learning scenario based on principles of cognitive apprenticeship and situated learning. The intervention was well received and the authors^28^ make recommendations for subsequent research and development that can help students develop the self‐efficacy, cognitive skills and critical thinking skills required for evidence‐based decision making.

Although brief, these descriptions show how each of these studies developed theoretical understanding and contributed to the improving of practice through the design and testing of interventions.

### 2.4 Key characteristics

In the same way that engineering design melds creative insights with pragmatic understanding and follows the best available theoretical principles derived from physics, materials science, aesthetics and other disciplines, EDR is a complex and multifaceted endeavour. The simultaneous pursuit of practical and scientific goals is central to the process, which can be characterised by five essential features. Educational design research is 'theorectically oriented' not only because it uses theory to ground design, but also because the design and development work is undertaken to contribute to broader scientific understanding. It is 'interventionist' because it is undertaken to engender productive change in a particular education context. It is 'collaborative' because it requires the expertise of multidisciplinary partnerships, including researchers and practitioners, but also often others (eg, subject matter specialists, software programmers and facilitators). It is 'responsively grounded' because its products are shaped by participant expertise, the literature and, especially, field testing. Finally, it is 'iterative' because it generally evolves through multiple cycles of design, development, testing and revision. Given these characteristics, it will come as no surprise that the overall duration of EDR studies is typically measured in years, rather than months.

Educational design research is not a methodology. This is important to mention because it clarifies that the methodological standards to which it should be held are no different from those of other kinds of research. The methodological rigour of EDR initiatives should therefore be judged using existing criteria for qualitative, quantitative or mixed‐methods studies (eg, reliability, validity, credibility, transferability, dependability, confirmability). Further, EDR leverages existing practices from the fields of design, sociology and education to shape participation and engagement.

## 3 CONDUCTING EDR

### 3.1 Modelling the process

**FIGURE 1:**
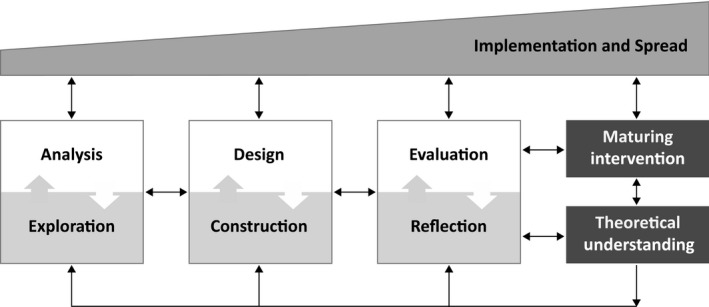
Generic model for conducting educational design research^7^

The present authors^7^ previously surveyed models for EDR, as well as for instruction design and curriculum development (eg, Ejersbo et al,^29^ Bannan‐Ritland,^30^ Wang and Hannafin^31^ ). Based on this analysis, we created a generic model for EDR, shown in Figure 1. This model shows a single, integrated research and development endeavour. It depicts the core elements of a flexible process that features the three main stages (described below), taking place in interaction with practice and yielding the dual outputs of knowledge and intervention. Each element of the model is discussed and examples are given.

Although additional information is given in the source publication,^7^ three main features bear mention here. First, the squares in Figure 1 denote three core phases, and the arrows between them indicate that the process is both iterative and flexible. During the 'analysis and exploration' phase, collaboration with practitioners is sought in order to shape a better understanding of the problem to be addressed. Research during this phase is typically informed by and contributes to theoretical understanding concerning the problem, context or stakeholders. During 'design and construction,' ideas about how the problem might be addressed tend to start off as rather large and vague, and gradually become refined, pruned and operationalised. Although this phase does not inherently involve empirical work, it does rely on and contribute to theoretical understanding about the (kind of) intervention, including its characteristics and underlying theory of action. During 'evaluation and reflection,' design ideas and prototype solutions are empirically investigated, and the findings are reflected upon, with the aim of refining (theoretical) understanding about if, how and why intervention features work. During this phase, research is shaped by and contributes to theoretical understanding about the (kind of) intervention or the responses it engenders. Across all phases, a blend of rational and creative mindsets is productive.

Second, the dual focus on theory and practice is made explicit through the rectangles, which represent the practical and scientific outputs, respectively. The practical solutions resulting from EDR can be educational products (eg, a multi‐user virtual learning game), processes (eg, a strategy for scaffolding medical student learning in a flipped classroom), programmes (eg, a series of workshops intended to help medical teachers develop more effective questioning strategies), or policies (eg, the designation of a minimum amount of one‐to‐one time for on‐site mentoring of interns). The theoretical understanding resulting from EDR can be used to describe, explain, predict or manipulate education phenomena. As noted previously, the theoretical understanding in design research underpins the design of the intervention, frames the scientific inquiry, and is advanced by findings generated through the empirical testing of the intervention.

Finally, the model demonstrates that attention to practical use through the trapezoid, which represents implementation and spread. It shows that interaction with practice is present from the start of the process, not as an afterthought, and increases over time. The bi‐directional arrows indicate that what happens in practice influences the ongoing core processes, as well as the ultimate outputs, and vice versa. Although not shown here, the professional development of those participating in the study (practitioners and researchers alike) is often a by‐product of the overall process and especially of the implementation work.

### 3.2 One example spanning all phases

**FIGURE 2:**
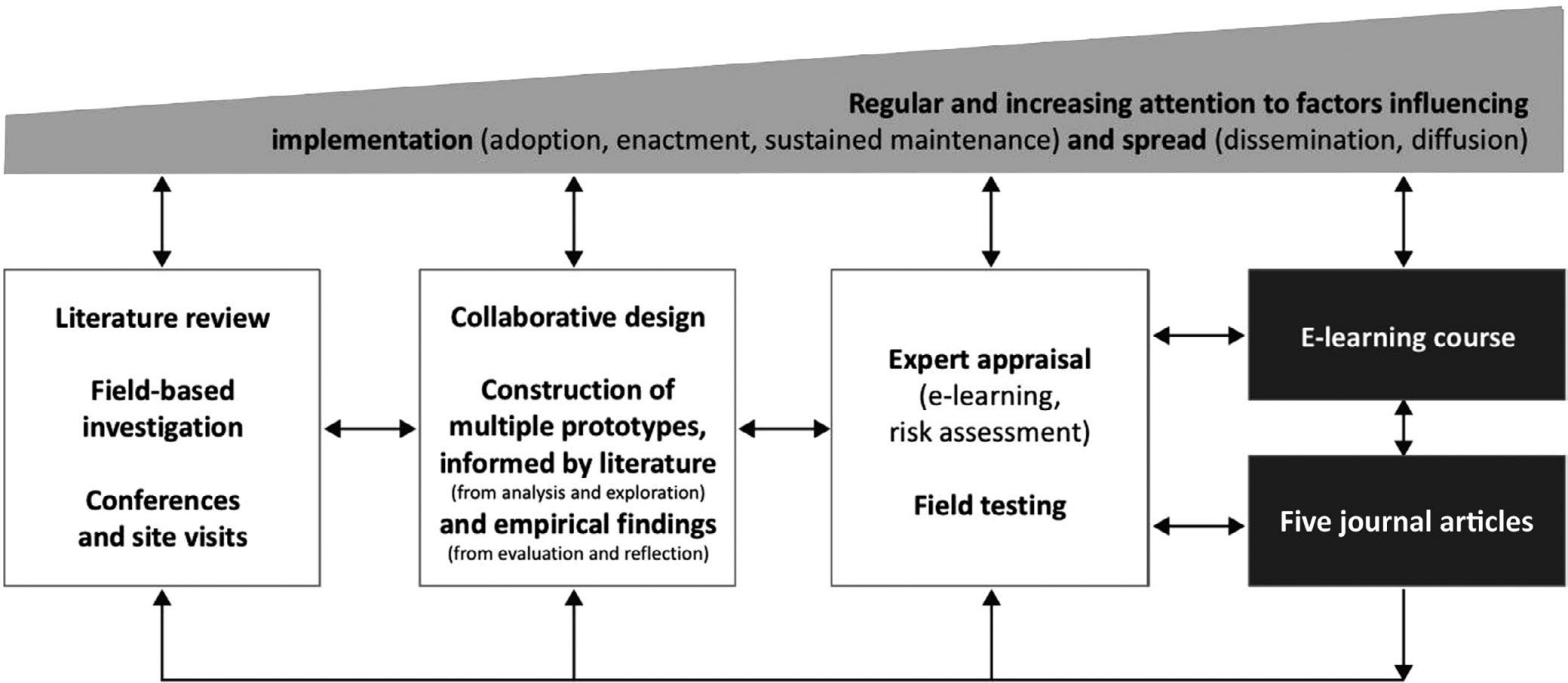
Overview of Vesper’s PhD study^32^ in light of the generic model for conducting design research in education^7^

To illustrate how this model comes to life, we briefly describe a previously published 4‐year PhD study, ^32^ which addressed a problem experienced by the World Health Organization (WHO). Namely, the WHO lacked a scalable, high‐quality training programme on the ‘cold chain’ that applies to the handling of vaccines and other pharmaceutical products that must be kept within the appropriate temperature range during shipping, storage and distribution. During analysis and exploration, Vesper^32^ conducted a literature review on relevant learning approaches, field‐based investigation to understand the state of the art of WHO e‐learning programmes, and participant observation of the existing cold chain training programme. During design and construction, Vesper developed and revised multiple training programme prototypes on the basis of the literature and empirical testing (ie, the outcomes of evaluation and reflection).^32^ During evaluation and reflection, Vesper^32^ used diverse strategies to investigate the various prototypes, including expert appraisal by e‐learning specialists, risk analysis by content experts, and field testing of a mature prototype with target users. From the very first analysis activities through to a final version of the course, attention was given to implementation and spread through close interaction with practitioners. In addition to the practical output (the e‐learning course and underlying design framework), which has subsequently been applied to the design of other online learning environments at the WHO,^33^ the scientific output is visible in the form of five journal articles, based on the investigation during analysis and exploration,^34^ design and construction,^35^ and evaluation and reflection^36^, ^37^, ^38^ phases, respectively. Figure 2 portrays this example in light of the generic model.

### 3.3 Multiple examples highlighting contributions from different phases

Across the shared characteristics of EDR, differences are also present. Some of the variation stems from the units of analysis, scope of implementation and nature of the subject areas addressed, as well as from the research domains and methodological traditions in which studies originate. The relative emphasis on each goal (solution development, new knowledge or both equally) can also wield strong influence on the design research process.

Here, different research reports^39^, ^40^, ^41^ are used to illustrate the variety of EDR conducted within the field of medical education. For each one, the problem addressed, the intervention developed, the knowledge created and the methods used are summarised in Table 2.

**TABLE 2:** Three examples demonstrating variations in educational design research

	Subramaniam et al^39^	Vandewaetere et al^40^	Bok et al^41^
Phase foregrounded in this article	Analysis and exploration	Design and construction	Evaluation and reflection
Problem addressed	Insufficient (research on) health literacy skills of adolescents, especially those from socioeconomically disadvantaged backgrounds	Insufficient (models for designing) learning scenarios for the acquisition of integrated competencies	Existing methods for developing and assessing workplace learning attend insufficiently to sustained professional competence
Intervention developed	HackHealth: an 8‐week, after‐school programme for children aged 10‐15 years	General practitioner learning modules (eg, 'Patient with Diabetes' and 'Young Child with Fever')	Competency‐based assessment programme for veterinary students
Knowledge created	Challenges encountered during health information seeking and related deficits in health information literacy	Exemplified guidelines on how to design an education programme based on whole‐task learning	Affordances and limitations of assessing longitudinal competency development through reflective and self‐directed learning activities
Methods used	Observations Interviews Focus groups Surveys Document analyses	Review and retrospective analysis of the steps taken to design and construct whole task learning	▪ Questionnaires ▪ Group interviews

The three examples described here^39^, ^40^, ^41^ illustrate how different types of research reports are published as sub‐components of larger EDR projects. Results from the analysis and exploration phase are highly visible in the article by Subramaniam et al,^39^ who used quantitative and qualitative methods in their design research to create, implement and revise HackHealth, an after‐school programme for health literacy that was targeted at adolescents from socioeconomically disadvantaged backgrounds. This particular sub‐study demonstrates how the implementation of the initial, literature‐based prototype provided the context for deeper analysis and exploration of the challenges encountered when completing various health‐related information activities. The findings extend beyond improving the HackHealth programme and hold important implications for working with this population, assessing and improving their health literacy skills, and designing instruction that stands to meet their needs.

The design and construction phase is central in the work of Vandewaetere et al,^40^ who describe the steps they took, and especially their underlying reasoning, in the course of building five learning modules for general practice students. Through authentic complex tasks such as caring for elderly people or handling patients with physically undefined symptoms, this work demonstrates how educators can address the development of integrated competencies, such as clinical reasoning, communication and health promotion. The module descriptions illustrate how principles of whole‐task learning and the 4C/ID (four‐component/instructional design) model can be applied, as well as also noting challenges and pitfalls in the educational innovation process, and thus offers guidance to others wishing to tackle similar challenges.

The evaluation and reflection processes and findings are foregrounded in the work of Bok et al,^41^ who investigated the implementation of a theory‐based assessment programme for veterinary medicine students. Their programme integrates learning and assessment by motivating and supporting students to seek, accumulate and learn from feedback in the workplace. They used quantitative and qualitative methods to explore the experiences of students and clinical supervisors. Their findings hold implications for revisions to their programme, as well as the development of similar initiatives. For example, peer feedback, social interaction and external guidance are crucial elements in this (kind of) programme, whereas the level of training required for portfolio judges and unintended student perceptions (namely, that even formative assessments are still summative) constitute challenges to be tackled head on.

## 4 ENHANCING EDR

### 4.1 Challenges and limitations

Phillips and Dolle,^42^ amongst others, have cautioned that the simultaneous pursuit of practical innovation and theory building is extremely ambitious and difficult. This partially stems from the fact that researchers pursuing design research work hand‐in‐hand with practitioners to grapple directly with the complex variation of real‐world education problems. Although it increases complexity, this collaboration can lead to the accomplishment of a third goal of EDR, that of professional development for all those involved. Such an ambitious agenda clearly brings its share of challenges.

Four types of challenges are commonly encountered when conducting EDR, and sensible researchers attempt to address them proactively. Conceptual challenges relate to understanding what EDR is (or is not) and the kinds of goals being pursued. From a methodological standpoint, EDR is challenging because, given the variety in the types of questions asked throughout a project’s lifecycle, it requires that researchers possess well‐rounded skills in a variety of methods. Communicating the processes and outcomes of EDR studies can be challenging because these studies are typically large and complex, and because their value to non‐stakeholders is not always articulated. A fourth set of challenges relates to political dimensions, often stemming from (implementing) the design, such as organisational policies and stakeholder dynamics. Table 3 presents an overview of the four common challenges: a) conceptual; b) methodological; c) communicative, and d) political that arise when conducting EDR. It also offers recommendations for addressing each.

**TABLE 3:** Common challenges and ways to mitigate them

Type	Challenge	Recommendation
Conceptual	Educational design research (EDR) (or design‐based research) is sometimes confused with research‐based design or action research	Clarify the theoretical contribution and especially the significance of the study for audiences not affiliated with the intervention context
	The long‐range goal and interim goals are related but different, a situation, which requires clarification	Noting that different questions are typically central at different stages of the work, map the overall theory of action and articulate underlying conjectures to clarify the focus of sub‐studies
Methodological	Methodological flexibility is required to answer multiple kinds of question (even within one trajectory)	Develop competence with a wide range of qualitative and quantitative methods, to enable selection based on purpose
	As with other forms of inquiry, educational design research (EDR) is easy to do poorly	Clearly meet the standards of rigour associated with the (qualitative, quantitative or mixed) methods being used
Communicative	The study seems too large or too complex to report in one (eg, article‐sized) chunk	Portray design projects as a collection of sub‐studies, reported separately, each making a significant contribution in its own right, and remain mindful that unnecessary discussion of the overall study can be distracting to the audience
	Because specific contexts are involved, the value to others (outside the research setting) seems limited	Situate the work as a (multiple) case study, clarifying the nature of the case and the frequency with which this phenomenon occurs, as well as also specifying salient details so that readers can ascertain if case‐to‐case generalisation is applicable
Political	Organisational barriers (eg, disciplinary barriers, human capacity) inhibit the work	Identify and focus work within the jurisdiction of change represented by the areas(s) within which the design project is able (by its own authority or through influence) to decide upon and implement change
	The power or opinions of stakeholders are valued or used in conflicting ways	Where possible, use facilitation skills and ethical guidelines to help parties negotiate priorities. In so doing, help all to see that deep and lasting change especially requires the commitment of those who will directly implement and sustain it

Every research approach has its limitations and EDR is no different in this regard. For those considering EDR, it is important to make informed choices. First, EDR requires close collaboration between (at least) researchers and practitioners.^43^ When this is not feasible or desirable, EDR ceases to be a viable option. Second, EDR connects theory, innovation and practice.^44^ When the development of one or more of these is not of high priority, EDR is not likely to be useful. Third, because it centres on creating productive change in practice, EDR requires substantial amounts of time.^45^ If the time available is measured in weeks (rather than months or years), EDR is not likely to be feasible.

Finally, given that EDR is fairly easy to do poorly and quite difficult to do well, a fourth limitation of EDR has less to do with the approach per se and more to do with the capacity of those conducting it. As with other ideas, the value of EDR lies not in its definition but in its realisation. ‘Design research is constituted within communities of practice that have certain characteristics of innovativeness, responsiveness to evidence, connectivity to basic science, and dedication to continual improvement.’^46^ In these trajectories, researchers must also fulfil the roles of designers and consultants, rendering this a highly challenging endeavour. McKenney and Brand‐Gruwel^47^ examined these three roles in light of each sub‐process of design research (analysis and exploration; design and construction; evaluation and reflection; implementation and spread). They articulate four foundational competencies that are required to enact each role, and offer guidelines for developing them. They note that 'empathy' is needed, for example, to explore (un)shared goals or become exposed to the incentives, motives and reward structures in different settings. 'Orchestration' helps to simultaneously attend to research framing, data collection, solution design, implementation, infrastructure woes and stakeholder ownership. Creative and analytical 'flexibility' supports the optimisation of the human and material resources available in ways that remain aligned with the goals of the instruction. Finally, 'social competence' refers to a robust repertoire of the interaction strategies needed to fulfil each role. Thus, conducting EDR is clearly a complex task. If this form of inquiry is to realise its potential contribution to the field of medical education, explicit attention must be given to the holistic development of design researcher capacity. As is the case with other forms of complex learning, this requires engaging with entire authentic design research projects. In deciding whether EDR is an appropriate fit for a given project, scholars are advised to consider the substantial and varied demands placed on researchers undertaking this kind of work.

### 4.2 CONCLUSIONS

Educational design research is of course no panacea. However, it does put the metaphorical brakes on solutionism because of its heightened attention to clarifying the nature of the problem before an educational intervention or solution is conceived. We believe that medical education faces many challenges that might be best addressed by synergistically pursuing both theoretical and development goals. For example, as Chen and Reeves^48^ argue, this approach could be used to:

- develop capacities to work effectively in increasingly fluid health care teams;
- cultivate skills to communicate in a culturally competent manner with patients and other health care professionals;
- prepare health care professionals for practice in a world that is increasingly infused with machine learning algorithms and robots;
- improve assessment protocols and feedback practices to promote competency‐based education, and
- enhance health care professionals’ clinical reasoning skills.

This article set out to discuss the nature and origins of EDR, how it is conducted, and what is needed to advance this kind of work. First, the approach was introduced, discussed in the light of related developments in the last century, as well as other contemporary approaches that strive towards both practical and theoretical goals, and characterised. Second, a generic model for conducting EDR was described and illustrated with multiple examples. Third, the challenges and limitations of this approach were considered. We conclude this article with a few words about next steps.

According to its website, this journal promises its readers ‘practically oriented and theory‐informed papers that emphasise empirical evidence and advancing the field.' As such, it would seem that EDR’s twin pursuits align well with the ambitions of *Medical Education*. If fluency with the approach has yet to be developed before this community shares its design studies (more), then collaboration with researchers in sister fields, who are more accustomed to design research (eg, those in the learning sciences, instructional design or educational technology) may be worth exploring. In so doing, it seems crucial to seek out like‐minded scholars who prioritise the giving of careful attention to ensuring descriptive and explanatory understanding of problems worth tackling before developing solutions. The contributions to this special issue on solutionism offer multiple starting points for doing just that.

## AUTHOR CONTRIBUTIONS

SMcK and TCR have been collaborating on the articulation of this approach for over a decade. This piece is based on that collaborative effort. SMcK drafted, revised and finalised the present text and figures. TCR edited and revised the present text. Both authors (SMck and TCR) developed the core ideas described in this paper, approved the final manuscript for submission, and agreed to be accountable for the work.

## 5 CONFLICTS OF INTEREST

Not applicable.

## 6 ETHICAL APPROVAL

Not applicable.
